# What Every Internist-Endocrinologist Should Know about Rare Genetic Syndromes in Order to Prevent Needless Diagnostics, Missed Diagnoses and Medical Complications: Five Years of ‘Internal Medicine for Rare Genetic Syndromes’

**DOI:** 10.3390/jcm10225457

**Published:** 2021-11-22

**Authors:** Anna G. W. Rosenberg, Minke R. A. Pater, Karlijn Pellikaan, Kirsten Davidse, Anja A. Kattentidt-Mouravieva, Rogier Kersseboom, Anja G. Bos-Roubos, Agnies van Eeghen, José M. C. Veen, Jiske J. van der Meulen, Nina van Aalst-van Wieringen, Franciska M. E. Hoekstra, Aart J. van der Lely, Laura C. G. de Graaff

**Affiliations:** 1 Department of Internal Medicine, Division of Endocrinology, Erasmus MC, University Medical Center Rotterdam, 3015 GD Rotterdam, The Netherlands; a.rosenberg@erasmusmc.nl (A.G.W.R.); m.pater@erasmusmc.nl (M.R.A.P.); k.pellikaan@erasmusmc.nl (K.P.); k.davidse@erasmusmc.nl (K.D.); f.hoekstra@rdgg.nl (F.M.E.H.); a.vanderlelij@erasmusmc.nl (A.J.v.d.L.); 2 Dutch Center of Reference for Prader-Willi Syndrome, 3015 GD Rotterdam, The Netherlands; 3 Stichting Zuidwester, 3241 LB Middelharnis, The Netherlands; a.kattentidt@zuidwester.org (A.A.K.-M.); r.kersseboom@zuidwester.org (R.K.); 4 Center of Excellence for Neuropsychiatry, Vincent van Gogh, 5803 DN Venray, The Netherlands; aroubos@vvgi.nl; 5 ‘s Heeren Loo, Care Group, 3818 LA Amersfoort, The Netherlands; a.m.vaneeghen@amsterdamumc.nl; 6 Department of Pediatrics, Amsterdam University Medical Center, 1105 AZ Amsterdam, The Netherlands; 7 Academic Center for Growth Disorders, Erasmus MC, University Medical Center Rotterdam, 3015 GD Rotterdam, The Netherlands; 8 ‘s Heeren Loo, Care Providing Agency, 6733 SC Wekerom, The Netherlands; josedietist@gmail.com (J.M.C.V.); jiske.van-der-meulen@sheerenloo.nl (J.J.v.d.M.); 9 Department of Physical Therapy, Erasmus MC, University Medical Center Rotterdam, 3015 GD Rotterdam, The Netherlands; n.vanaalst-vanwieringen@erasmusmc.nl; 10 Department of Internal Medicine, Reinier de Graaf Hospital, 2625 AD Delft, The Netherlands; 11 ENCORE—Dutch Center of Reference for Neurodevelopmental Disorders, 3015 GD Rotterdam, The Netherlands; 12 Dutch Center of Reference for Turner Syndrome, 3015 GD Rotterdam, The Netherlands; 13 Dutch Center of Reference for Disorders of Sex Development, 3015 GD Rotterdam, The Netherlands

**Keywords:** syndrome, intellectual disability, missed diagnosis, medical overuse, internal medicine, multidisciplinary care

## Abstract

Patients with complex rare genetic syndromes (CRGS) have combined medical problems affecting multiple organ systems. Pediatric multidisciplinary (MD) care has improved life expectancy, however, transfer to internal medicine is hindered by the lack of adequate MD care for adults. We have launched an MD outpatient clinic providing syndrome-specific care for adults with CRGS, which, to our knowledge, is the first one worldwide in the field of internal medicine. Between 2015 and 2020, we have treated 720 adults with over 60 syndromes. Eighty-nine percent of the syndromes were associated with endocrine problems. We describe case series of missed diagnoses and patients who had undergone extensive diagnostic testing for symptoms that could actually be explained by their syndrome. Based on our experiences and review of the literature, we provide an algorithm for the clinical approach of health problems in CRGS adults. We conclude that missed diagnoses and needless invasive tests seem common in CRGS adults. Due to the increased life expectancy, an increasing number of patients with CRGS will transfer to adult endocrinology. Internist-endocrinologists (in training) should be aware of their special needs and medical pitfalls of CRGS will help prevent the burden of unnecessary diagnostics and under- and overtreatment.

## 1. Introduction

Patients with complex genetic syndromes (CRGS), by definition, have combined medical problems affecting multiple organ systems [1,2,3]. Intellectual disability and challenging behavior are often part of the syndrome. During the last decades, healthcare for children with CRGS has significantly improved. Most children with CRGS receive multidisciplinary (MD) and specialized medical care in which usually three to four medical specialists are involved [4]. Whereas in the past, many genetic syndromes were associated with premature death, this improvement of medical care has increased life expectancy and a growing number of children with CRGS now reach adult age [5,6]. Although the complexity of the manifestations of CRGS generally persists into adulthood, proper syndrome-specific MD hospital care is rarely available for adults with CRGS. While multiple organ systems are usually affected, specialists with a broad scope, like internists and endocrinologists, are seldomly involved. After transfer from pediatric to adult medical care, patients and their parents often report fragmented care of poor quality instead of adequate and integrated health management [7,8,9,10,11,12,13,14,15]. Furthermore, information on the natural course and medical guidelines are often lacking for these ‘new adults’. In our experience, the lack of adequate information can lead to undiagnosed health problems, suboptimal treatment, and painful and expensive complications. Therefore, pediatricians and intellectual disability physicians (ID physicians) have expressed the urgent need for adequate, specialized, MD ternary hospital care for adults with CRGS [16,17]. To address this need, we have launched a specialized MD outpatients’ clinic (MOPC) within the Department of Internal Medicine, Division of Endocrinology, specially designed for adults with CRGS. The MD team consists of an internist-endocrinologist, neuropsychologist, ID physician, clinical geneticist, dietitian for intellectual disability, physiotherapist, and nurse practitioner. As MD care for adults with CRGS is not available elsewhere in the Netherlands, our center serves as a ternary referral center. This results in the clustering of relatively large numbers of patients with rare syndromes in our center, which gives us the unique opportunity to study their medical needs and syndrome-specific manifestations. In this article, we describe our experience of the first five years of ‘Internal Medicine-Endocrinology for Complex Rare Genetic Syndromes’. We report the most frequent syndrome-specific complaints and provide an illustrative case series of patients who underwent unnecessary (invasive) diagnostics tests for symptoms and complaints that were either direct (primary somatic manifestations) or indirect consequences of the syndrome. Based on our experience and previous literature, we provide an overview of syndrome-specific endocrine and non-endocrine manifestations and provide practical advice for diagnostics and treatment. By sharing our experience, we aim to show the importance of specialized MD care for adults with CRGS. Our data will help other internist-endocrinologists prepare for the increasing numbers of patients with CRGS that are now reaching adult age and transferring to internal medicine/adult endocrinology. Creating awareness of the special needs of adults with CRGS and of the pitfalls in diagnosis and treatment will likely prevent unnecessary interventions and prevent painful and expensive complications in this vulnerable patient group.

## 2. Materials and Methods

Approval for this retrospective study was waived by the local Medical Ethics Committee of the Erasmus University Medical Center Rotterdam (EMCR, Rotterdam, The Netherlands). Informed consent from individual patients or their legal representatives was obtained for the case series. We reviewed the medical files of all adults who visited the Center for CRGS at the Endocrinology Unit of the Department of Internal Medicine of the EMCR between April 2015 and December 2020. Although Klinefelter syndrome (KS) does not meet the European definition of rare diseases (1:2000 persons affected [18]), KS is included in this article as adults with KS also visited our center for CRGS. As adult manifestations are unknown for most CRGS and as history taking may be hindered by ID, pre-visit medical questionnaires and systematic health screening are standard procedures of routine patient care of our center. The systematic screening consists of a medical questionnaire, structured interview (including use of medication), a complete physical examination, a review of the medical records, biochemical measurements, and, if indicated and feasible, additional tests. Part of the data on Prader-Willi syndrome (PWS) was previously published [19].

### 2.1. Genetic Diagnosis

Genetic confirmation was either collected from the referring physician or was performed at our medical center to confirm the clinical diagnosis.

### 2.2. Medical Questionnaire

As part of regular patient care, patients or their primary caregivers filled out a questionnaire before visiting the MOPC. The questionnaire included items on the patient’s medical history, medication, family history, symptoms of disease, physical complaints, behavioral challenges, and social aspects (work, school, relationship, and living situation). The items “symptoms of disease”, “physical complaints”, and “behavioral challenges” were rated on a 5-point Likert scale (1 = rarely or never, 2 = not often and/or not severe, 3 = quite often and/or quite severe, 4 = often and/or severe, 5 = very often and/or very severe). A score of 3 or higher was considered clinically relevant and was further examined during the visit. The composition of the MD team was based on the answers that were provided in the pre-visit questionnaire.

### 2.3. Biochemical Analysis

During the visit, blood samples were taken for general screening, including evaluation of the hematopoietic system, kidney function, liver enzymes, glucose metabolism, fat metabolism, thyroid function, gonadal function, and vitamin D status.

### 2.4. Additional Tests

We screened for hypertension at the first visit to our MOPC. In case of a blood pressure above 140/90 mmHg, the measurement was repeated and if it was still elevated, a 30 min blood pressure measurement was performed. Patients were diagnosed with borderline hypertension if the first blood pressure measurement was above 140/90 mmHg and the repeated blood pressure measurements were around 140/90 mmHg. Other additional tests that we often perform at our center, but are beyond the scope of this article, include dual-energy X-ray absorptiometry in case of clinical suspicion of osteoporosis or osteopenia and polygraphy or polysomnography in case of clinical suspicion of sleep apnea.

### 2.5. Literature Search

Literature was searched in several databases (MEDLINE (via ALL OVID), Embase, and Cochrane Central Register of Controlled Trials) for clinical manifestations of the syndromes that we encountered at our MOPC. The full search strategy is available upon request.

### 2.6. Data Analysis

Data were analyzed with IBM SPSS version 25 and R version 3.6.0. Data are presented as the mean ± standard deviation for continuous data and percentage (number) for categorical data. We clustered “daytime sleepiness” and “general muscle weakness” with “tiredness”, as in our experience, patients use the term “tiredness” also to express “daytime sleepiness” and “general (muscle) weakness”. The term “fatigue” represents the clustered variable. “Abdominal complaints” were defined as the presence of constipation, diarrhea, and/or abdominal pain. Frequencies of complaints are reported separately for the syndromes for which syndrome-specific questionnaires were available, namely Prader-Willi syndrome (PWS), Neurofibromatosis type 1 (NF1), KS, and “other CRGS”.

## 3. Results

During the first 5 years of the MOPC, we have treated 720 adults, with 61 different CRGS (Table 1). In 41 patients, there was a clinical suspicion of a genetic syndrome (due to the combination of dysmorphic features, intellectual disabilities, and/or dysfunction of multiple organs) but the underlying genetic etiology was unknown.

**Figure 1:**
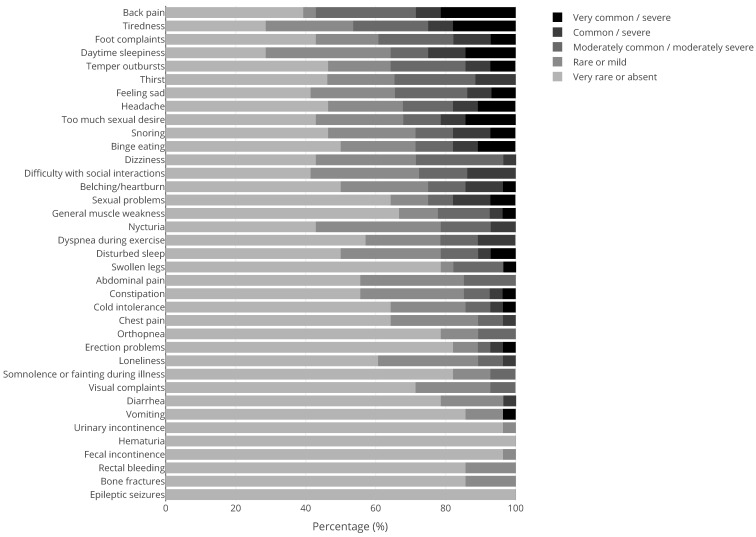
Physical complaints reported by (caregivers of) adults with Klinefelter syndrome (N = 29). The tones of grey represent the scores 1 (rarely or never; lightest shade) to 5 (very often and/or very severe; darkest shade), see also the Section 2. The dree darkest tones of grey are considered clinically relevant (score ≥ 3).

**Figure 2:**
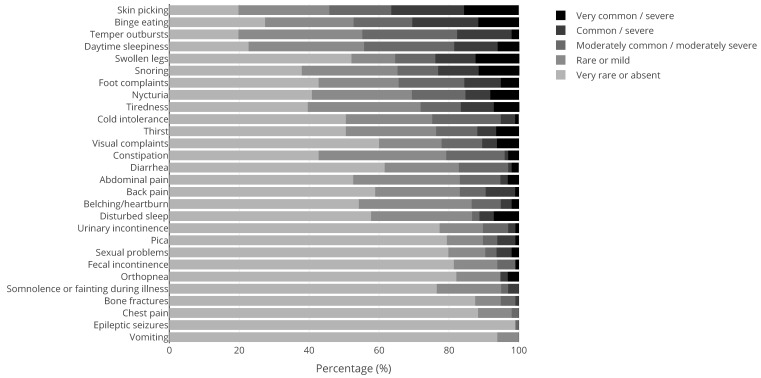
Physical complaints reported by (caregivers of) adults with Prader-Willi syndrome (N = 100). The tones of grey represent the scores 1 (rarely or never; lightest shade) to 5 (very often and/or very severe; darkest shade), see also the Section 2. The dree darkest tones of grey are considered clinically relevant (score ≥ 3).

**Figure 3:**
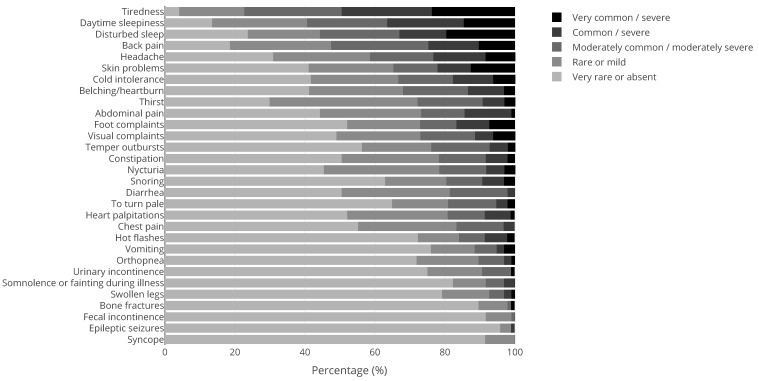
Physical complaints reported by (caregivers of) adults with Neurofibromatosis type 1 (N = 97). The tones of grey represent the scores 1 (rarely or never; lightest shade) to 5 (very often and/or very severe; darkest shade), see also the Section 2. The dree darkest tones of grey are considered clinically relevant (score ≥ 3).

**Figure 4:**
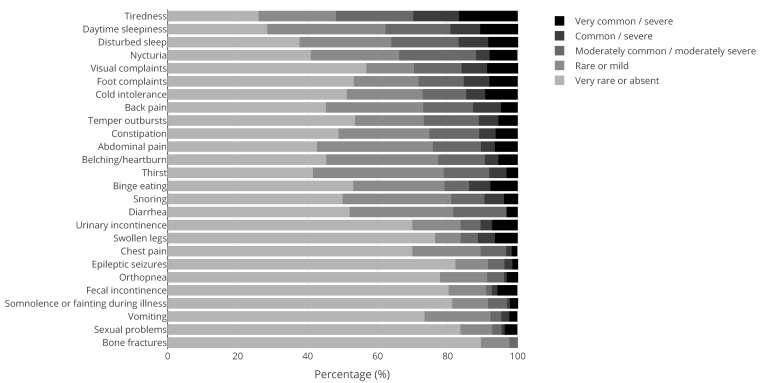
Physical complaints reported by (caregivers of) adults with rare genetic syndromes other than Prader-Willi syndrome, Neurofibromatosis type, or Klinefelter syndrome (N = 143). The tones of grey represent the scores 1 (rarely or never; lightest shade) to 5 (very often and/or very severe; darkest shade), see also the Section 2. The dree darkest tones of grey are considered clinically relevant (score ≥ 3).

Apart from 41 men with KS and 184 women with Turner syndrome, 43% (N = 211) of our patient population were male and 57% (N = 284) were female. The mean ± SD age was 35.8 ± 13.3 years. The medical questionnaire was filled out by 369 patients of which 100 PWS patients, 97 NF1 patients, 29 KS patients, and 143 patients with other genetic CRGS. Figure 1, Figure 2, Figure 3 and Figure 4 show the physical complaints reported by the patient and/or their primary caregivers in the medical questionnaire. The most frequent complaints were fatigue (60%; N = 223) and abdominal complaints (37%; N = 135); the prevalence of these complaints differed between the different CRGS (Figure 1, Figure 2, Figure 3 and Figure 4). The population was characterized by polypharmacy and the most frequently used drugs were psychotropic drugs (22%; N = 56 out 251) and pain medication (22%, N = 25 out 112).

Table 2 shows the clinical manifestations of all CRGS seen in our center during the study period. Eighty-nine percent of the syndromes were associated with endocrine problems and 72% with intellectual disability. High blood pressure was common; among the 720 patients who visited our center, hypertension was present in 20% (N = 142 out 678) of the patients; 2% (N = 11 out 678) had borderline hypertension.

**Figure 5:**
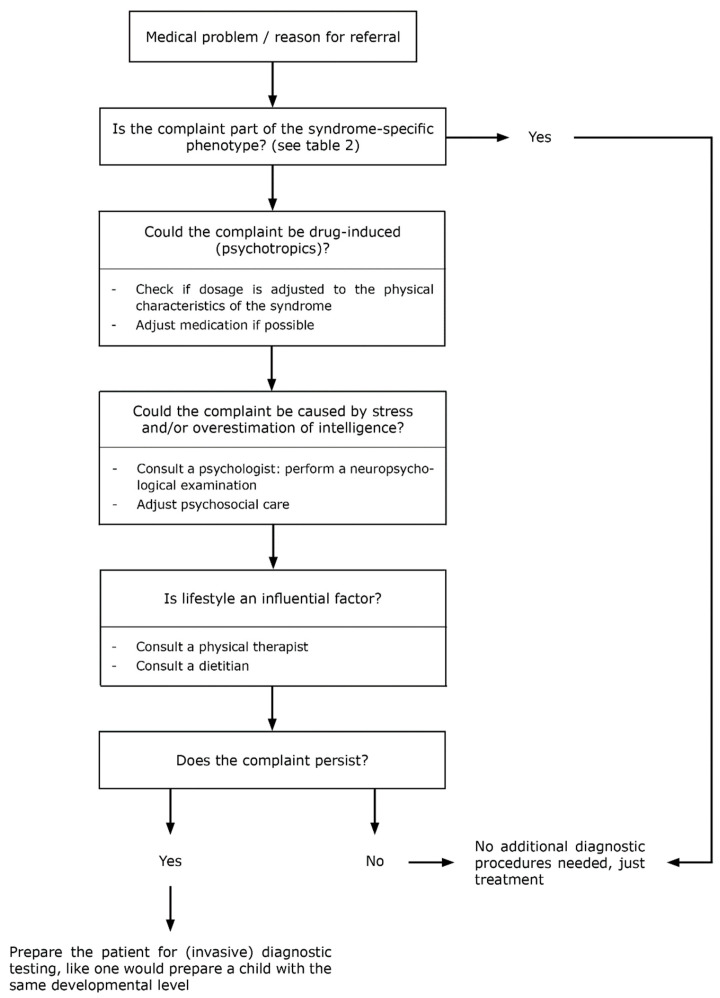
Algorithm for the approach to medical problems in patients with CRGS.

**Figure 6:**
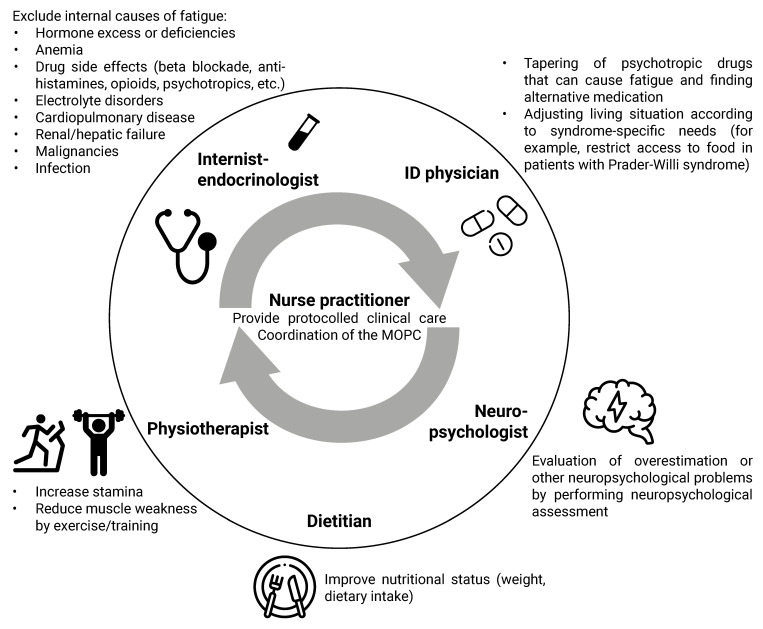
Multidisciplinary approach in our clinic to the patient with a complex rare genetic syndrome and fatigue. Abbreviations: ID, intellectual disability; MOPC, multidisciplinary outpatients’ clinic. The figure was designed with resources from flaticon.com (accessed on 30 September 2021) (Freepik, Smashicons).

**Figure 7:**
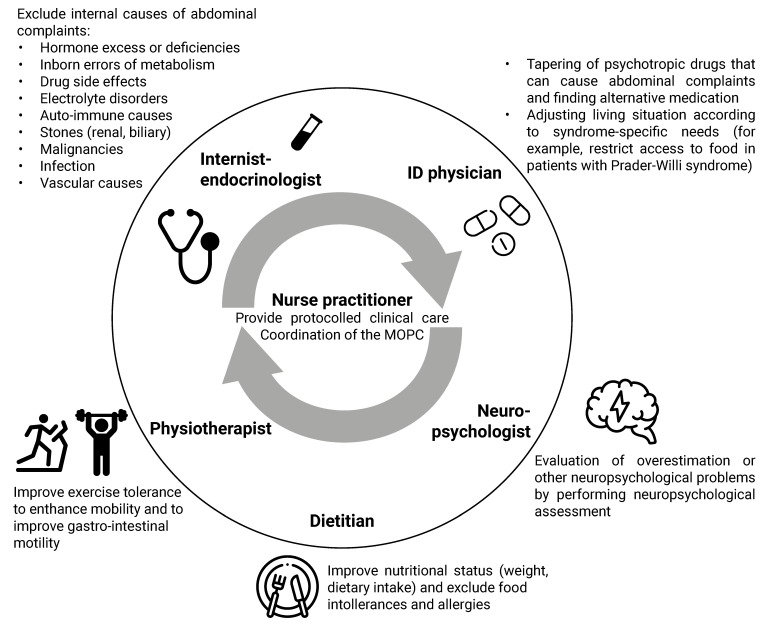
Multidisciplinary approach in our clinic to the patient with a complex rare genetic syndrome and abdominal complaints. Abbreviations: ID, intellectual disability; MOPC, multidisciplinary outpatients’ clinic. The figure was designed with resources from flaticon.com (accessed on 30 September 2021) (Freepik, Smashicons).

**Figure 8:**
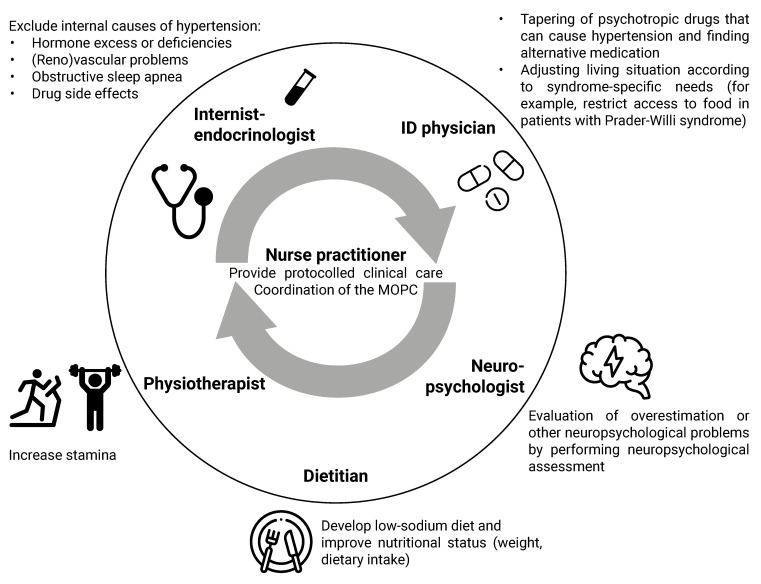
Multidisciplinary approach in our clinic to the patient with a complex rare genetic syndrome and hypertension. Abbreviations: ID, intellectual disability; MOPC, multidisciplinary outpatients’ clinic. The figure was designed with resources from flaticon.com (accessed on 30 September 2021) (Freepik, Smashicons).

We developed an algorithm for the approach to medical problems in adults with CRGS (Figure 5) and provide an overview of the MD approach to the most common medical problems (fatigue, abdominal complaints, and hypertension) among adults with CRGS (Figure 6, Figure 7 and Figure 8).

Among the 720 patients who visited our center, we found several patients with missed diagnoses and patients who had undergone unnecessary diagnostic procedures for symptoms that actually could be explained by their syndrome. The most illustrative cases are described below.

### 3.1. Case Series: Missed Diagnoses

#### 3.1.1. Case 1: Untreated Diabetes Mellitus in a Patient with PWS

A 28-year-old male with PWS was referred to our center because of fatigue, weight gain (BMI: 35 kg/m^2^), and polydipsia. He was seen by a pediatrician until he was 18 years of age. Growth hormone treatment was initiated during puberty and was stopped when the patient reached adult height. He visited the general practitioner (GP) because of fatigue and polydipsia. The GP assumed that polydipsia, like hyperphagia, was part of the PWS-spectrum and assumed that fatigue was inherent to PWS. Therefore, he did not perform any further diagnostic testing. The patients’ mother was worried about her son and asked for a referral to our center. Our systematic screening revealed that his polydipsia was caused by untreated diabetes mellitus. Furthermore, we found untreated hypogonadism and vitamin D deficiency, which possibly also contributed to his fatigue. We provided the patient and caregivers with diet and exercise recommendations, followed by metformin and liraglutide (sulfonylurea derivatives were not administered due to the possible side effect of weight gain). After normalization of blood glucose levels, polydipsia was no longer a complaint. At follow-up visits, fatigue had also disappeared.

#### 3.1.2. Case 2: Untreated Hypothyroidism, Hypogonadism and Vitamin D Deficiency in a Patient with PWS

A 23-year-old male with PWS visited the GP because of weight gain (BMI: 32.5 kg/m^2^), fatigue, passive behavior, and poor exercise tolerance. The GP performed a physical examination and biochemical analysis, including TSH to screen for hypothyroidism. TSH was normal; other hormone or vitamin deficiencies were not included in the GP’s screening. Unaware of the fact that hypothyroidism in PWS often has a central origin, the GP concluded that hypothyroidism was absent and that symptoms of fatigue and passive behavior were part of the PWS-phenotype. Due to persistent complaints, the patient visited our MOPC. As a normal TSH does not rule out central hypothyroidism associated with PWS, we measured free T4 and found severe untreated hypothyroidism. Our screening also revealed undiagnosed hypogonadism and vitamin D deficiency. After replacement with thyroid hormone, testosterone, and vitamin D, exercise tolerance improved and the patient lost 30 kg of weight (BMI: 23.4 kg/m^2^). Fatigue was no longer present and his passive behavior disappeared.

#### 3.1.3. Case 3: Untreated Heart Failure in a Patient with PWS

A 28-year-old woman with PWS suffered from weight gain and leg edema. NT-proBNP (N-terminal pro-brain natriuretic peptide) levels measured by the internist were normal and cardiac ultrasound detected no abnormalities. Unaware of the fact that cardiac ultrasound and NT-proBNP are unreliable in PWS (NT-proBNP can be false negative in up to 15% of obese patients [230]), the internist concluded that the patient’s symptoms were not caused by any cardiac problem. Shortly after visiting the internist, the patient developed severe shortness of breath and was admitted to the intensive care unit with respiratory insufficiency due to cardiac failure. After adequate treatment of the heart failure, the patient is doing well.

### 3.2. Case Series: Overtreatment

#### 3.2.1. Case 4: Hypersexuality in a Patient with Cockayne Syndrome

A 20-year-old male with Cockayne syndrome was referred to our MOPC because of hypersexual behavior. The patient was on the waiting list to undergo a bilateral orchidectomy because of his hypersexuality with inappropriate sexual behavior. His medication list revealed that he was using methylphenidate. The treating physician at that time assumed that hypersexual behavior was part of the Cockayne phenotype. Hypersexuality is, however, not part of the spectrum of Cockayne syndrome. We, therefore, searched for alternative explanations, such as drug side effects. As increased libido can be a side effect of methylphenidate, this was tapered. His hypersexuality disappeared and the bilateral orchidectomy was canceled.

#### 3.2.2. Case 5: Overtreatment with Hydrocortisone in a Patient with PWS

A 30-year-old male with PWS was referred to our MOPC due to progressive obesity. A few years earlier, he had been diagnosed with central adrenal insufficiency (CAI), based on low morning cortisol found by an internist in a regional hospital. The patient had been taking daily hydrocortisone ever since and had gained 2 kg per year. He presented at our clinic with a BMI of 32 kg/m^2^. As CAI is extremely rare in adults with PWS (even in patients with low morning cortisol [164]), we gradually tapered the hydrocortisone. One year after stopping hydrocortisone, BMI had returned to normal. A metyrapone test confirmed the absence of CAI.

### 3.3. Case Series: Unnecessary Diagnostic Tests

#### 3.3.1. Case 6: Depression and Behavioral Problems in a Patient with PWS

A 47-year-old male with PWS was referred to our clinic due to frequent falls and apathy, with the request to rule out underlying somatic pathology. The patient had visited a neurologist to rule out neurologic pathology. A brain CT had not revealed any abnormalities. He had also visited a cardiologist who had performed an electrocardiogram, cardiac ultrasound, and blood tests, which were all normal.

In the past, the GP had prescribed high dosages of pipamperon for temper outbursts, after which the patient had developed epileptic seizures. Subsequently, a neurologist had prescribed levetiracetam to treat the epileptic seizures. As notable side effects of pipamperon and levetiracetam include sleepiness and depression [231], we suspected that his apathy could be caused by the high dosage of psychotropic drugs. As the initial temper outbursts appeared to be triggered by stress, we advised adapting the (intensive) daycare program, after which temper outbursts were no longer a problem. Pipamperon and levetiracetam were gradually tapered, after which his apathy disappeared.

#### 3.3.2. Case 7: Unnecessary Invasive Testing (Colonoscopy and Magnetic Resonance Cholangio-Pancreatography (MRCP)) in a Patient with Williams-Beuren Syndrome

A 52-year-old woman with Williams-Beuren Syndrome (WBS) was referred to our MOPC by the clinical geneticist. She had an intellectual disability with a developmental age of an 8-year-old child. Her medical history revealed hypertension for which she was taking three different antihypertensive drugs. When asking for stress as a potential cause of her hypertension, the patient became emotional and reported having undergone several invasive diagnostic tests which she had experienced as very traumatic. She had undergone a colonoscopy to find the cause of her diarrhea. She had also undergone an MRCP to find the cause for mild liver dysfunction. As our screening revealed undiagnosed and untreated diabetes mellitus type II, we concluded that her mild liver dysfunction was probably caused by diabetes mellitus related non-alcoholic fatty liver disease. Diarrhea turned out to be a side effect of the medication she was using (pancreatin). Colonoscopy and MRCP had been carried out according to regular procedures for adults with normal intelligence. The patient had not been able to process the information provided before and during these invasive procedures. As patients with WBS have remarkably strong verbal skills, over-estimation of cognitive capacities is a common pitfall in patients with WBS [232]. The physician, unaware of the impact that these invasive tests could have on the mental health of the patient, had not linked the hypertension to previous invasive procedures. We referred the patient to the psychologist for psychological assistance and trauma treatment, after which the anti-hypertensive drugs could be successfully tapered.

## 4. Discussion

We report our five-year experience of ‘internal medicine-endocrinology for adults with complex rare genetic syndromes’. Illustrated by a population overview and case series, we show that missed diagnoses, undertreatment, overtreatment, and unnecessary diagnostic tests seem common among adults with CRGS. We hypothesize that this is largely due to a lack of syndrome-specific knowledge among internists-endocrinologists and GPs.

The lack of syndrome knowledge is not surprising, as internists-endocrinologists hardly encountered any adults with rare genetic syndromes until recently. However, due to increased life expectancy, a growing number of patients with CRGS is now reaching adult age. Therefore, it is important to raise awareness of syndrome-specific manifestations, medical needs, and diagnostic pitfalls of this vulnerable population. The most frequent physical complaints in our population were fatigue (60%) and abdominal complaints (37%). Furthermore, many patients had hypertension (20%). These medical problems were much more frequent in adults with CRGS than in the general population, where fatigue is present in 31% [233], abdominal complaints in 2–4% [234], and hypertension in 5–10% [235]. However, it must be noted that patients are only referred to our center in case of (suspected) pathology in the internal medicine domain. This might result in a higher prevalence of hypertension, fatigue, and abdominal complaints.

For this paper, we mainly focused on the recognition of and approach to physical complaints in adults with CRGS. However, early diagnosis of complications associated with the different syndromes, as displayed in Table 2, is also an important aspect of clinical care for adults with CRGS. In our center, we screen for these complications with a systematic health screening [19]. Further research should also focus on how to improve the early diagnosis of complications associated with CRGS. Furthermore, for the case series, the number of cases about PWS is higher than one would expect, based on the incidence of PWS (1:16,000–21,000 live births [236,237]). Since our center is the only Reference Center for adults with PWS in the Netherlands, a relatively high number of adults with PWS are treated at our center, which resulted in more cases of Prader-Willi syndrome.

Based on our experience with more than seven hundred CRGS adults, we provide two main clinical recommendations which will help other internist-endocrinologists to provide good clinical care to this vulnerable patient population.

### 4.1. Multidisciplinary Approach

Our first recommendation is to treat adults with CRGS in a multidisciplinary setting. As CRGS patients often have multiple health problems simultaneously, a multidisciplinary team should ideally consist of an internist-endocrinologist, psychologist, nurse practitioner, dietitian, physiotherapist, clinical geneticist, and ID physician. If it is not possible to include an ID physician in the team, a behavioral expert or psychiatrist should be included to address behavioral issues. Ideally, a nurse practitioner should be part of the multidisciplinary team, to coordinate the MD care and provide protocolled clinical care for (relatively) high-prevalence syndromes.

Due to the multi-organ involvement of many of the syndromes, it is important to have direct lines of communication with other specialists, such as cardiologists, gynecologists, urologists, neurologists, and dermatologists, and include them in the MD team when needed.

The MD approach of the most common complaints in our cohort (fatigue and abdominal complaints) and hypertension is shown in Figure 6, Figure 7 and Figure 8.

Although organizing MD care might seem expensive, it will probably be cost-efficient in the long term. The social-economic burden of rare diseases in Europe, including PWS, is high [238,239]. One way of reducing the high annual economic burden (estimated to be around EUR 60.000 per patient) for patients with PWS [240] is improving clinical care by establishing MOPCs. Several studies have shown that MOPCs are cost-effective for diseases such as heart failure, kidney disease, and diabetes mellitus [241,242]. Although to our knowledge no studies were performed to date on the cost-effectiveness of MOPCs for CRGS, they will likely reduce costs in a similar manner by preventing overtreatment, unnecessary diagnostic tests, and medical complications. Although the annual economic burden will likely decrease, the total economic burden might be similar, due to the increased number of life-years as a result of the improved MD care. However, this will probably be compensated by the expected improved quality of life in adults with CRGS.

A number of adult dedicated clinics have been established in order to treat adults with specific ‘higher prevalence’ syndromes, such as Down syndrome [243,244] and Turner syndrome [245,246,247,248]. However, MD care for patients with (ultra-)rare syndromes was not available yet. Therefore, in our center, we have organized MD care for all adults with CRGS, including extremely rare syndromes. To our knowledge, our multidisciplinary center for CRGS is the first worldwide, in the field of internal medicine-endocrinology. It must be noted, however, that besides establishing an MD center for CRGS, the transition from pediatric to adult care should be optimized to prevent fragmented and inadequate care.

The multidisciplinary aspect of the healthcare we provide, not only has medical advantages; it is also much appreciated by the patients and their caregivers as shown by a satisfaction survey that was performed during the first year of the MOPC. Meeting all necessary disciplines during one visit saves them time, effort, stress, and money that would otherwise be spent on traveling to separate hospital visits.

### 4.2. Clinical Algorithm for the Approach to Physical Symptoms

Our second recommendation is to use a clinical algorithm we have developed for the approach to physical symptoms in adults with CRGS (Figure 5). This approach differs from regular internal medicine patient care due to syndrome-specific health problems, but also due to the increased prevalence of polypharmacy and stress and unfavorable lifestyle related to intellectual disability and/or challenging behavior often present in patients with CRGS.

#### 4.2.1. Syndrome-Specific Complaints

For any complaint the patient presents with, the internist-endocrinologist or nurse practitioner should always first check whether the problem could be inherent to the syndrome (Table 2).

#### 4.2.2. Drug Side Effects

If a complaint or symptom is not part of the syndrome, one should check whether it could be a drug-related side effect. As polypharmacy is common in adults with CRGS, drug-related side effects should be considered as the cause of any potential complaint. Especially psychotropic drugs are known to cause fatigue, abdominal complaints, and hypertension [249]. When the presenting complaint or symptom is indeed caused by psychotropic drugs, tapering the dose, or finding alternative medication should always be done in consultation with an ID physician or psychiatrist.

#### 4.2.3. Chronic Stress

Once drug-related side effects have been excluded, one should consider whether the presenting complaint or symptom might be caused by chronic stress. In our experience, overestimation of cognitive capacities is a frequent cause of chronic stress and stress-related physical complaints in patients with (unrecognized) intellectual disabilities. Especially in syndromes where verbal intelligence exceeds performance intelligence (for example in PWS or WBS), patients are easily overestimated. If overestimation is indeed suspected, neuropsychologists can perform neuropsychologic assessments to assess the actual cognitive capacities. The ID-physician can help residential homes in adjusting the support and living situation of the patient to their IQ and syndrome-specific needs. For example, restriction of access to food can prevent a lot of stress in patients with PWS who are (due to their hyperphagia) continuously foraging and bargaining for food. The behavioral therapist can subsequently provide training to the caregivers to improve patient-caregiver interaction and address any potential stress-related issues. Moreover, if the patient has no intellectual disability, chronic stress can occur due to syndrome-related impaired executive functions or other psychological and contextual factors. In that case, the support of a neuropsychologist or behavioral therapist is also warranted.

#### 4.2.4. Lifestyle

If the complaint can be improved by lifestyle or other contextual modifications, a dietitian or physiotherapist (specialized in patients with intellectual disability when necessary) should be consulted. Especially in large residential homes, physical activity and nutrition are often inadequate [250]. In the case of physical or intellectual disabilities, lifestyle interventions should be customized to the capacities of the individual patient [251].

#### 4.2.5. Additional Diagnostic Testing

Only if the symptom is not part of the syndrome, nor related to polypharmacy, intellectual disability, behavioral factors, and/or lifestyle, or persists despite adequate treatment, underlying organic causes should be excluded by diagnostic tests. If diagnostic procedures are needed, adults with intellectual disabilities should be informed in a way that is appropriate for their developmental age, to prevent traumatic experiences.

## 5. Conclusions

Our experience with 720 adults with CRGS illustrates that the medical management of CRGS adults poses significant challenges for healthcare professionals. The complex medical and neuropsychiatric comorbidity can lead to missed diagnoses on the one hand, and to unnecessary invasive procedures on the other hand. As more and more CRGS patients are now reaching adult age and making the transfer to internal medicine/adult endocrinology, internist-endocrinologists should be aware of the medical pitfalls and the special needs of adults with CRGS.

Based on our findings and the literature, we recommend treating adults with CRGS in a multidisciplinary team and using the clinical algorithm we have developed for the approach to physical symptoms in adults with CRGS.

As knowledge about syndrome-specific health problems is crucial to prevent the personal and financial burden of unnecessary diagnostics and under- and overtreatment, we believe that education about syndrome-specific health problems in adults with CRGS should be part of the internist-endocrinologists’ medical training.

## Figures and Tables

**Table 1:** Overview of complex rare genetic syndromes seen in our multidisciplinary outpatient clinic.

Syndrome	N	Syndrome	N	Syndrome	N
Albright hereditary osteodystrophy	<5	Dandy-Walker syndrome	<5	Ring chromosome 21	<5
Allan-Herndon-Dudley syndrome	<5	DiGeorge syndrome (22q11.2 deletion)	8	Saethre-Chotzen syndrome	<5
Alström syndrome	<5	Disorders of Sex Development ^1^	18	Say-Barber-Biesecker-Young-Simpson syndrome (KAT6B mutation)	<5
Angelman syndrome	<5	Down syndrome (trisomy 21)	<5	Sifrim-Hitz-Weiss syndrome	<5
Axenfeld-Rieger syndrome	<5	Hypogonadotropic hypogonadism with anosmia (Kallmann syndrome)	6	Silver-Russell syndrome	5
Bardet-Biedl syndrome	5	Hypogonadotropic hypogonadism without anosmia (Kiss1R mutation)	<5	Smith-Lemli-Opitz syndrome	<5
Bloom syndrome	<5	Jacobsen syndrome	<5	Smith-Magenis syndrome	<5
Börjeson-Forssman-Lehmann syndrome	<5	Joubert syndrome	<5	Sotos-like syndrome	<5
CAMK2A variants	<5	JS-X syndrome	<5	Tatton-Brown-Rahman syndrome	<5
CHARGE syndrome	10	Kabuki syndrome	<5	TBL1X mutation	<5
CHD8 syndrome	<5	KAT6A syndrome	<5	Tetra-X syndrome	<5
Chromosome 1q21 deletion syndrome	<5	Klinefelter syndrome	41	Triple-X syndrome	<5
Chromosome 1q25-32 deletion	<5	L1CAM mutation	<5	TRPV4 mutation	<5
Chromosome 16p11.2 deletion syndrome	<5	Myhre syndrome	<5	Tuberous sclerosis complex	49
Chromosome 16p3.11 deletion syndrome	<5	Neurofibromatosis type 1	120	Turner syndrome	184
Cockayne syndrome	<5	Noonan syndrome	9	Williams-Beuren syndrome	10
Congenital adrenal hyperplasia	11	PNPLA6 gene mutation	<5	45,X/46,XY mixed gonadal dysgenesis	<5
Cornelia de Lange syndrome	<5	PTEN hamartoma tumor syndrome	<5	48,XXXY syndrome	<5
Costello (like) syndrome	<5	Prader-Willi like syndrome	9	48,XXYY syndrome	<5
Cri-du-chat syndrome	<5	Prader-Willi syndrome	135	Unknown syndrome	41
CTNNB1 syndrome	<5	Rett syndrome	<5	**Total**	**720**

Abbreviations: N, number of patients. As some syndromes only occurred in a very small number of patients, a threshold of 5 was chosen for the sake of patient privacy. ^1^ This includes complete androgen insensitivity syndrome, Leydig cell hypoplasia, partial androgen insensitivity syndrome, 5α-reductase deficiency, 17β-hydroxysteroid dehydrogenase type 3 deficiency, 17–20 desmolase deficiency, and partial 17-hydroxylase deficiency, and 46,XX + SRY. The bold distinguishes the total number of patients clearly from the syndromes.

**Table 2:** Clinical manifestations of complex rare genetic disorders seen in our center since 2015.

	Endocrine Manifestations	Internal Medicine—Other	Other Disciplines
Albright hereditary osteodystrophy [20,21]	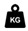 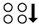 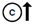 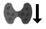 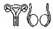 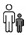	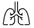 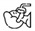	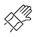 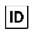 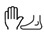 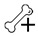 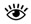 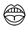
Allan-Herndon- Dudley syndrome [22,23]	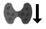 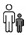	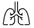	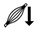 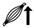 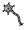 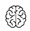 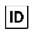 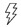 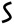 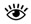
Alström syndrome [24,25]	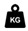 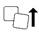 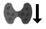 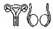 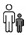	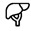 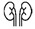 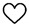 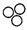 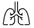	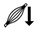 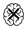 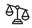 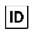 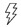 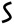 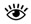 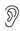 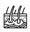 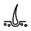
Angelman syndrome [26,27]	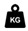 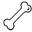	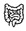 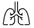	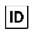 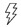 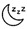 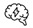 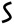 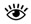
Axenfeld-Rieger syndrome [28,29]		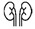 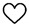	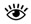 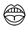 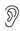
Bardet-Biedl syndrome [30,31,32,33,34]	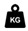 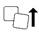 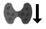 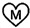 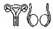 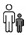 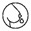 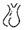	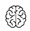 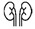 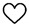 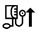 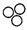	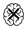 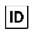 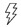 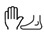 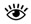
Bloom syndrome [35]	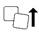 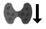 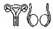 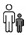	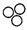 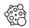 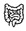 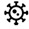	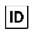 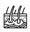
Börjeson-Forssman-Lehmann syndrome [36,37]	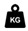 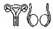 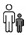 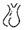 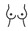		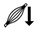 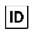 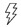 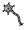 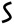 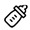 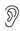
CAMK2A variants [38,39]	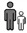 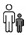	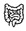 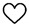	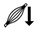 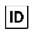 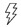 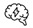 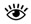
CHARGE syndrome [40,41,42,43,44,45,46,47,48,49,50,51]	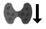 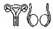 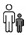 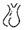 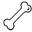	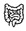 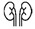 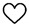 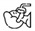	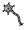 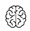 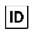 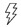 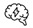 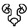 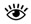 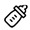 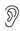 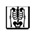 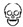
CHD8 syndrome [52,53,54]	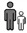	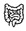	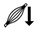 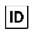 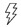 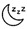 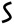 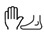 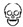 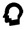
Chromosome 1q21 deletion syndrome [55,56,57]	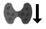 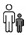	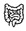 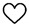	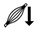 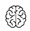 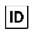 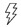 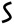 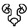 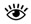 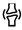 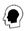 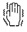 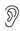
Chromosome 1q25-32 deletion [58,59]	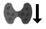 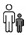		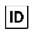 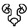 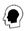
Chromosome 16p11.2 deletion syndrome [60,61,62]	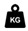 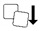	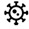	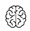 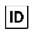 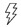 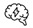
Chromosome 16p13.11 deletion syndrome [63,64,65]		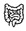	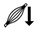 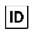 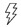 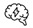 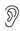 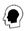
Cockayne syndrome [66,67,68,69,70]	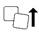 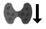 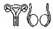 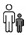	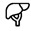 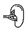 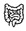 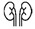 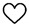 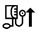 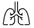	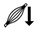 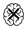 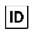 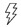 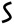 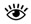 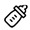 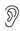 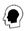 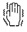 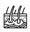 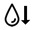
Congenital adrenal hyperplasia [71,72]	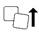 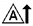 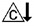 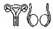	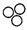 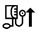	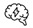 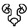
Cornelia de Lange syndrome [73,74,75,76,77]	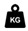 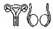 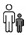	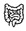 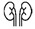 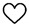 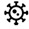	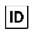 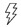 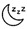 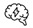 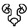 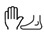 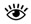 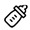 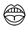 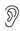 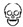 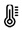 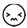
Costello (like) syndrome [78,79,80]	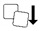 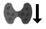 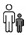 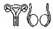 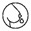	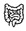 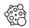 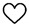 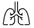 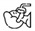	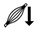 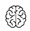 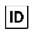 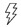 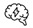 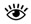 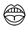 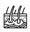 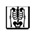
Cri-du-chat syndrome [81,82,83,84]		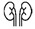 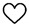	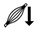 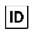 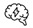 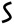 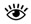 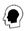
CTNNB1 syndrome (NEDSDV syndrome) [85,86,87]		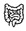 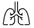	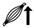 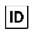 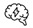 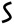 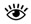 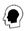 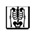
Dandy-Walker syndrome [88,89]	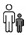	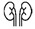 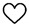	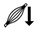 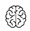 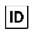 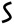 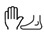 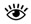 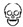
DiGeorge syndrome (22q11.2 deletion) [90,91,92,93,94,95,96,97,98,99,100,101,102,103,104,105]	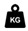 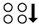 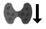 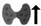 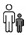	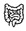 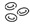 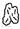 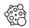 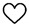 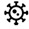 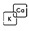	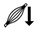 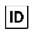 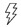 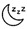 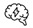 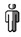 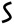 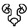 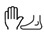 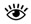 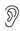 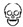
Disorders of Sex Development ^1^ [106,107]	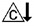 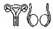	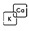 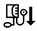	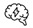
Down syndrome (trisomy 21) [108,109,110,111,112,113]	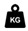 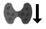 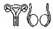 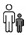 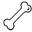	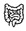 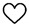 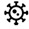 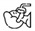	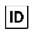 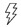 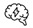 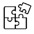 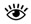 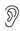
Hypogonadotropic hypogonadism with anosmia (Kallmann syndrome) [114,115]	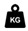 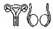 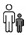	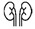	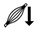 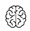 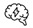 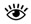 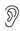 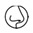 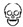
Hypogonadotropic hypogonadism without anosmia (Kiss 1R mutation) [116,117]	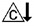 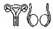 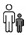		
Jacobsen syndrome (11q terminal deletion syndrome) [118,119,120,121]	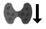 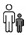	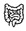 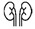 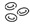 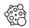 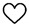 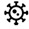	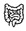 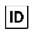 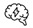 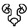 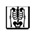
Joubert syndrome [122,123]	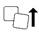 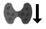 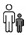 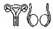 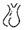	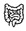 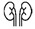 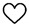 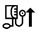 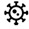	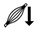 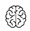 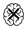 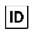 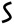 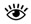
JS-X syndrome [124]			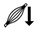 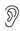 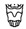
Kabuki syndrome [125,126,127,128,129]	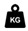 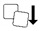 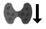 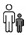 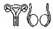	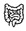 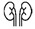 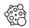 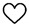 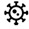	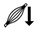 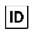 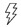 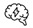 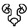 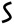 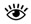 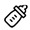 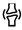 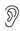 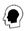
KAT6A syndrome [130,131]	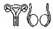	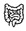 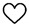 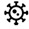	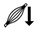 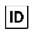 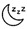 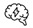 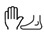 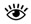 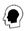
Klinefelter syndrome [132,133,134,135,136,137,138]	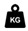 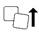 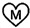 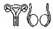 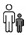 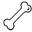 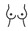	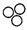 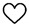 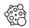	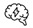
L1CAM mutation [139,140,141]	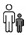	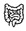 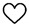	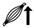 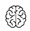 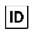 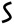 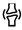
Myhre syndrome [142,143,144]	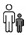 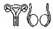	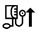	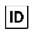 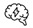 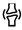 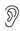 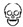
Neurofibromatosis type 1 [145,146,147,148,149,150,151,152,153,154]	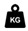 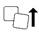 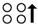 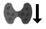 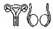 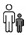 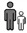 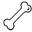	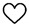 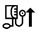 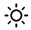 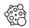	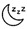 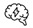 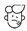 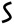 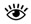 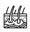
Noonan syndrome [155,156,157]	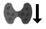 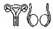 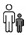	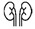 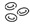 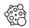 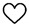	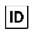 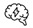 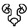 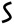 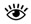 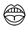 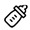 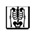
PNPLA6 gene mutation [158]	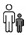 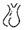		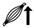 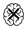 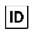 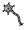 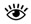 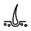
PTEN hamartoma tumor syndrome [159,160,161]	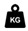 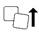	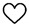 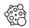	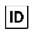 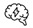 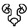 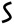 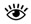 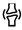 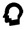 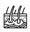 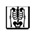
Prader-Willi (like) Syndrome [19,162,163,164,165,166,167,168,169,170,171]	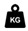 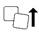 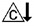 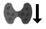 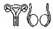 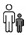 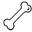	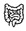 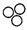 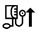 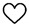 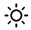 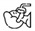	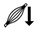 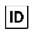 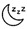 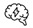 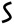 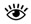 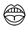 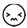
Rett syndrome [172,173,174,175,176]	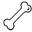	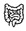 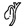 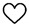	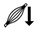 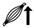 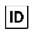 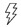 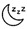 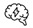 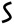 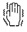
Ring chromosome 21 [177,178]	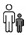	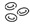 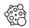 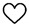 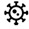	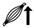 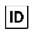 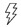 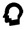
Saethre-Chotzen syndrome [179,180,181]	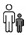	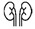 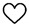 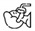	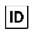 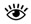 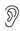 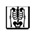 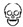
Say-Barber-Biesecker-Young-Simpson syndrome (KAT6B mutation) [182,183,184]	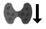	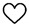	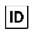 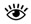 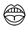 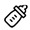 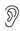 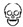
Sifrim-Hitz-Weiss Syndrome [185,186]	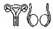 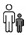	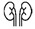 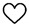	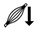 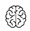 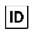 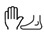 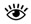 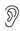 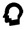 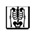
Silver-Russell syndrome [82,187,188,189,190,191]	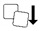 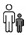	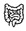 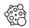 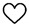	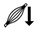 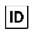 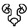 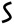 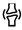 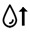
Smith-Lemli-Opitz syndrome [192,193,194,195,196]	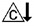 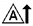 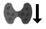	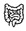 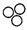 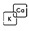 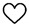 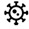	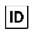 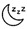 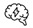 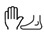 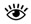 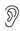 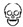 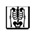
Smith-Magenis Syndrome [82,197]	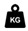 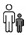	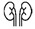 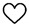	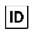 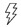 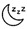 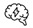 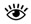 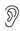 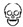 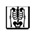 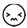
Sotos-like syndrome [198,199,200,201]	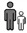	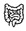 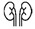 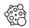 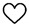	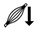 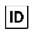 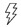 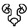 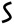 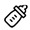 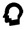
Tatton-Brown- Rahman syndrome [202,203]	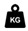 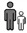	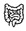 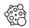 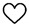 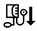 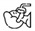	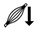 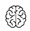 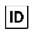 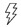 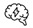 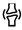 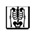
TBL1X mutation [204,205,206]	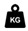 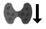	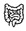	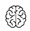 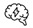 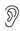
Tetra-X syndrome (48,XXXX) [207,208,209,210,211,212]	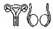 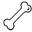 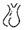	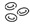 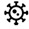	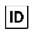 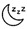 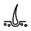 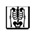
Triple-X syndrome (47,XXX) [213,214]	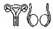 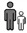	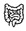 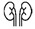	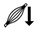 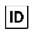 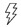 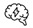 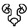
TRPV4 mutation [215]	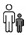 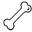	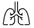	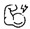 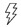 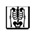
Tuberous sclerosis complex [216,217]		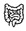 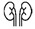 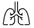 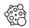	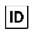 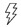 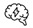 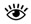 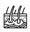
Turner syndrome [218,219]	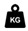 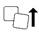 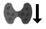 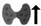 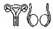 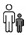 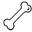	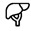 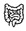 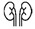 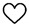 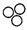 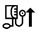 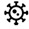 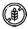	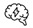 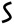 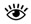 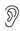 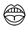 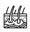
Williams-Beuren syndrome [220,221,222,223]	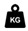 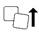 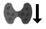 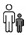 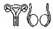 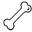	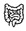 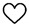 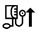 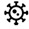 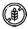 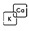	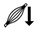 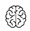 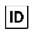 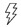 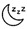 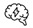 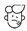 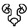 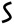 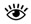 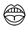 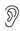 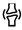
45,X/46,XY mixed gonadal dysgenesis [224,225]	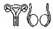 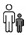	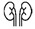 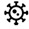	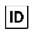
48,XXXY syndrome [226]	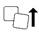 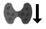 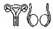 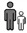 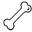	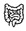 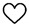 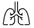 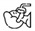	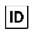 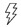 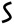 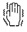
48,XXYY syndrome [226,227,228,229]	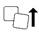 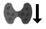 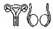 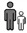 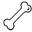	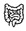 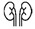 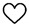 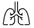 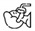	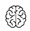 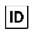 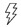 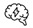 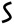 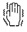

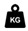
obesity 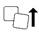 diabetes mellitus 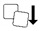 hypoglycemia 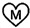 metabolic syndrome 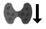 hypothyroidism 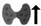 hyperthyroidism 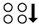 (pseudo)hypoparathyroidism 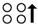 hyperparathyroidism 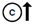 hypercalcitoninemia 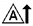 hyperaldosteronism 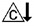 impaired cortisol synthesis 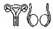 disturbed gonadal axis 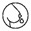 hyperprolactinemia 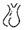 hypopituitarism/pituitary anomalies 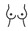 gynecomastia 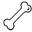 osteopenia/osteoporosis 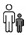 short stature/growth hormone deficiency 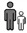 tall stature/overgrowth 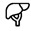 hepatic disease/anomalies 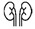 renal disease/anomalies 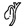 gallbladder disease 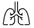 pulmonary problems 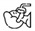 obstructive sleep apnea 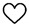 cardio(vascular) disease/anomalies 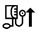 hypertension 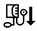 hypotension 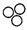 dyslipidemia 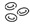 hematologic anomalies 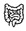 gastrointestinal problems/anomalies 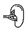 splenomegalie 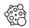 increased risk of malignancies 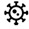 immune problems/anomalies 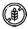 celiac disease 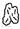 thymic hypoplasia 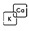 electrolyte disorders 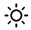 vitamin D deficiency 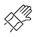 carpal tunnel syndrome 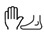 limb anomalies 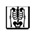 skeletal anomalies 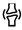 joint problems 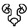 urogenital tract anomalies 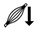 hypotonia/low muscle mass/muscle atrophy 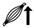 hypertonia 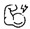 neuromuscular problems 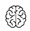 brain anomalies 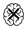 ataxia 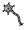 hypomyelination/cranial nerve anomalies/(poly)neuropathy 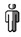 hemiparesis 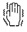 tremor/parkinsonism 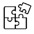 Alzheimer disease 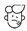 increased risk of stroke 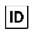 intellectual disability/developmental disorders 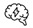 psychological problems/challenging behavior 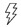 epilepsy/seizures 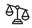 poor balance 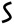 scoliosis 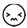 decreased pain sensitivity 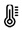 temperature intolerance 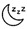 sleeping problems 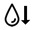 decreased sweat production 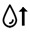 increased sweat production 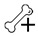 ectopic ossification 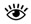 visual problems/anomalies 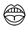 oral problems/anomalies 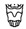 laryngeal obstruction 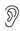 hearing problems/anomalies 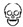 craniofacial anomalies 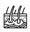 skin anomalies 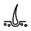 hair problems 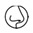 hyposmia/anosmia 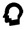 macrocephaly 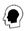 microcephaly 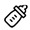 feeding difficulties. A more detailed overview of the clinical manifestations of these complex rare genetic disorders is given in the Supplementary Data, Table S1. In reality, some manifestations might have a similar prevalence as in the general population, because of publication bias. Although the literature was thoroughly searched, the overview might be incomplete. ^1^ There are many different types of Disorders of Sex Development. Therefore, we advise checking the specific type of disorder in the literature for the specific clinical manifestations. Icons were derived from flaticon.com (accessed on 30 September 2021) (Freepik, DinosoftLabs, Pixel perfect, Flat Icons, Smashicons, Good Ware, smalllikeart, Vitaly Gorbachev, monkik, Kiranshastry, Eucalyp, surang, lcongeek26, Chanut) and from the Noun Project (icondfield, VectorsPoint, tezar tantular) on 30 September 2021.

## Data Availability

Some or all datasets generated during and/or analyzed during the current study are not publicly available but are available from the corresponding author on reasonable request.

## Supplementary Materials

The following are available online at https://www.mdpi.com/article/10.3390/jcm10225457/s1, Table S1: Clinical manifestations of complex rare genetic disorders seen in our center since 2015.
