# Supplementary material for: What Every Internist-Endocrinologist Should Know about Rare Genetic Syndromes in Order to Prevent Needless Diagnostics, Missed Diagnoses and Medical Complications: Five Years of ‘Internal Medicine for Rare Genetic Syndromes’
Source: J Clin Med. 2021 Nov 22;10(22):5457. doi: 10.3390/jcm10225457 (PMC8622899; doi:10.3390/jcm10225457)
Supplement: Supplementary file 1 [file jcm-10-05457-s001.zip › jcm-1456221-supplementary.pdf]

Supplementary data

Title: What every internist-endocrinologist should know about rare genetic syndromes in order to prevent needless diagnostics, missed diagnoses and medical complications: Five years of 'Internal Medicine for Rare Genetic Syndromes'

Authors: Anna G.W. Rosenberg, Minke R.A. Pater, Karlijn Pellikaan, Kirsten Davidse, Anja. A. Kattentidt-Mouravieva, Rogier Kersseboom, Anja G. Bos-Roubos, Agnies van Eeghen, José M.C. Veen, Jiske J. van der Meulen, Nina van Aalst-van Wieringen, Franciska M.E. Hoekstra, Aart J. van der Lely, Laura C.G. de Graaff

Corresponding author: Laura C.G. de Graaff, MD, PhD; Department of Internal Medicine-Endocrinology, Erasmus University Medical Center. Dr. Molewaterplein 40, 3015 GD Rotterdam, the Netherlands. E-mail: l.degraaff@erasmusmc.nl

Table S1. Clinical manifestations of complex rare genetic disorders seen in our center since 2015

	Endocrine manifestations	Internal medicine – Other	Other disciplines
Albright hereditary osteodystrophy	• Obesity (42)<br>• Parathyroid hormone resistance (42)<br>• Hypercalcitoninemia (42)<br>• Thyroid-stimulating hormone resistance, unexplained congenital hypothyroidism (42)<br>• Gonadotropin resistance (hypogonadism) (42)<br>• Growth hormone deficiency (42)<br>• Advanced skeletal maturation (42)<br>• Short stature (42)	• Asthma (42)<br>• Sleep apnea (42)	• Carpal tunnel syndrome (43)<br>• Neurological problems, including impaired cognition and intellectual disability (42)<br>• Acrodysostosis (42)<br>• Ectopic ossifications (42)<br>• Cataract (42)<br>• Ear infections (42)<br>• Oral complications (42)<br>• Cryptorchidism (42)
Allan-Herndon-Dudley syndrome	• Dysthyroidism (high free T3, normal to low-normal serum T4, upper-normal TSH) (44)<br>• Short stature (44)	• Pneumopathies (45)	• Hypotonia (44)<br>• Spasticity (45)<br>• Dystonia (45)<br>• Hypomyelination (45)<br>• Brain atrophy (45)<br>• Muscular hypoplasia (44)<br>• Intellectual disability (44)<br>• Seizures (44)<br>• Scoliosis (44)<br>• Ocular abnormalities (44)
Alström syndrome	• Obesity (46)<br>• Diabetes mellitus type II (46)<br>• Growth hormone deficiency(47)<br>• Hypogonadism (46)<br>• Hypothyroidism (46)<br>• Hyperthyroidism (46)<br>• Advanced skeletal maturation (46)	• Hepatic disease (46)<br>• Renal dysfunction (46)<br>• Cardiomyopathy (46)<br>• Hypertriglyceridemia (46)<br>• Pulmonary failure (47)	• Visual problems (46)<br>• Hearing impairment (46)<br>• Mild ataxia (46)<br>• Hypotonia (46)<br>• Poor balance (46)<br>• Seizures (46)<br>• Acanthosis nigricans (47)<br>• Intellectual disability/developmental delay (47)<br>• Scoliosis (47)

			• Alopecia (46)
Angelman syndrome	• Obesity (48)<br>• Osteopenia/osteoporosis (48)	• Gastroesophageal reflux (49)<br>• Constipation (48)<br>• Pneumonia (48)	• Intellectual disability (48)<br>• Epilepsy (48)<br>• Sleep disturbances (48)<br>• Behavioral problems (48)<br>• Visual problems (48)<br>• Scoliosis (48)<br>• Seizures (48)
Axenfeld-Rieger syndrome		• Kidney abnormalities (50)<br>• Heart defects (50)	• Dental abnormalities (51)<br>• Ocular abnormalities, including glaucoma (50)<br>• Abnormal brain development (50)<br>• Hearing loss (50)
Bardet-Biedl syndrome	• Non insulin-dependent diabetes mellitus (52)<br>• Nephrogenic diabetes mellitus (53)<br>• Metabolic syndrome (53)<br>• Hypogonadism (52)<br>• Growth hormone deficiency (54)<br>• Hyperprolactinemia (54)<br>• Structural pituitary abnormalities (54)<br>• Obesity (52)<br>• Subclinical hypothyroidism (53)<br>• Short stature (52)	• Hepatic fibrosis (52)<br>• Hypertension (53)<br>• Hypertriglyceridemia (53)<br>• Hypercholesterolemia (52)<br>• Renal dysfunction (52)	• Intellectual disability (52)<br>• Ocular abnormalities (52)<br>• Ataxia (55)<br>• Spasticity (55)<br>• Congenital heart defects (53,56)<br>• Polydactyly and dystrophic extremities (52)
Bloom syndrome	• Abnormal carbohydrate metabolism (57)<br>• Dyslipidemia (57)<br>• Hypothyroidism (57)	• Malignancies (57)<br>• Diarrhea (57)<br>• Gastroesophageal reflux (57)	• Skin abnormalities (57)<br>• Early motor and speech delays (57)

	• Diabetes mellitus type II (57)<br>• Infertility/subfertility (57)<br>• Short stature (57)	• Vomiting (57)<br>• Immune abnormalities (57)	
Börjeson-Forssman-Lehmann syndrome	• Obesity (58)<br>• Hypopituitarism (58)<br>• Gynaecomastia (58)<br>• Hypogonadism (59)<br>• Short stature (58)		• Intellectual disability (58)<br>• Feeding difficulties (58)<br>• Hypotonia (58)<br>• Polyneuropathy (59)<br>• Epilepsy (58)<br>• Perthes disease (58)<br>• Hearing problems (58)<br>• Scoliosis (58)
CAMK2A variants	• Overgrowth (60)<br>• Growth delay (61)	• Cardiac abnormalities (60)<br>• Gastrointestinal problems (60)	• Intellectual disability (60)<br>• Behavioral problems (60)<br>• Hypotonia (61)<br>• Seizures/convulsions (61)<br>• Visual problems (61)<br>• Cryptorchidism (60)
CHARGE syndrome	• Growth hormone deficiency (62)<br>• Hypogonadotropic hypogonadism (62,63)<br>• Thyroid-stimulating hormone deficiency (62)<br>• ACTH deficiency (62)<br>• Structural pituitary abnormalities (62)<br>• Osteoporosis (64)	• Gastrointestinal problems, including constipation, vomiting, difficulty swallowing and gastroesophageal reflux (65,66)<br>• Cardiovascular malformations (67,68)<br>• Obstructive sleep apnea (69)<br>• Renal abnormalities (68)	• Intellectual disability (67)<br>• Cranial nerve abnormalities (70)<br>• Brain abnormalities (68)<br>• Seizures (71)<br>• Autism spectrum disorder (68)<br>• Ocular abnormalities (71)<br>• Ear abnormalities, including hearing loss (68,72)<br>• Cleft palate (68)<br>• Genital/urinary tract abnormalities (68,73)<br>• Feeding difficulties (65)<br>• Skeletal abnormalities (68)
CHD8 syndrome	• Overgrowth (74)	• Constipation (75)	• Intellectual disability (74)<br>• Autism spectrum disorder/autism traits (74)<br>• Hypotonia (74,76)<br>• Seizures (74)<br>• Scoliosis (74)

			• Clinodactyly (74)<br>• Umbilical hernia (74)<br>• Glabellar hemangioma (74)<br>• Motor delay (76)<br>• Macrocephaly (75)<br>• Sleep disorder (76)
Chromosome 1q21 deletion syndrome	• Hypothyroidism (77)<br>• Short stature (77)	• Gastric ulcers (77)<br>• Cardiac abnormalities (77,78)	• Intellectual disability (77,78)<br>• Impaired motor function (77)<br>• ADHD (77)<br>• Cataracts (78)<br>• Joint laxity (78)<br>• Hypotonia (78)<br>• Epilepsy/seizures (78)<br>• Scoliosis (77)<br>• Autism spectrum disorder (77)<br>• Microcephaly (77)<br>• Tremor (77)<br>• Sensorineural deafness (79)<br>• Genitourinary abnormalities (79)<br>• Brain malformations (79)
Chromosome 1q25-32 deletion	• Hypothyroidism (80)<br>• Growth hormone deficiency (80)<br>• Short stature (80)		• Intellectual disability (80,81)<br>• Microcephaly (80)<br>• Genital abnormalities (80)
Chromosome 16p11.2 deletion syndrome	• Obesity (82,83)<br>• Hyperinsulinemic hypoglycaemia (82)	• Possibly increased risk of severe combined immunodeficiency (84)	• Intellectual disability (83)<br>• Motor/developmental delay (83)<br>• Language disorder (82)<br>• Autism spectrum disorder (82,83)<br>• Psychological problems (83)<br>• Epilepsy/seizures (82,83)<br>• Structural abnormalities of the central nervous system (82)<br>• ADHD (83)
Chromosome 16p13.11 deletion syndrome		• Constipation (85)	• Developmental delay (86)<br>• Motor delay (86)<br>• Abnormal behavior (86)<br>• Hypotonia (86)

			• Hearing loss (86)<br>• Microcephaly (86)<br>• Epilepsy (87)<br>• Schizophrenia (85)
Cockayne syndrome	• Hypogonadism (88)<br>• Abnormal glucose metabolism (89)<br>• Growth failure (89)<br>• Hypothyroidism (89)	• Hypertension (89)<br>• Dilated cardiomyopathy (90)<br>• Aortic dilatation (91)<br>• Gastroesophageal reflux (89)<br>• Hepatic dysfunction (89)<br>• Splenomegaly (92)<br>• Renal problems (89)<br>• Respiratory infections (89)<br>• Restrictive lung disease (89)<br>• Asthma (89)	• Intellectual disability (92)<br>• Motor problems (88)<br>• Microcephaly (89)<br>• Ataxia (88)<br>• Intracranial calcification (88)<br>• Dermatologic abnormalities (89)<br>• Sensorineural deafness (89)<br>• Seizures (89)<br>• Tremor (89)<br>• Feeding difficulties (89)<br>• Poor peripheral circulation (89)<br>• Visual problems (90)<br>• Scoliosis (92)<br>• Muscle atrophy (92)<br>• Decreased production of sweat (88)
Congenital adrenal hyperplasia	• Inadequate aldosterone production (93)<br>• Impaired cortisol synthesis (93)<br>• Insulin resistance (94)<br>• Diabetes mellitus type II (94)<br>• Decreased fertility (93)	• Salt wasting (93)<br>• Failure to thrive (93)<br>• Hypovolemia/shock (93)<br>• Hypertension (94)<br>• Increased carotid intima thickness (94)<br>• Hyperlipidemia (94)	• Abnormal development of external genitalia (93)<br>• Psychosocial problems (93)
Cornelia de Lange syndrome	• Hypogonadism (95)<br>• Growth failure (95)<br>• Obesity (96)	• Gastroesophageal reflux (96)<br>• Constipation (96)<br>• Renal dysfunction (95)<br>• Aspiration (95)<br>• Congenital heart defects (95)<br>• Antibody deficiency (95)	• Intellectual disability (95)<br>• Epilepsy / seizures (95,96)<br>• Temperature intolerance (95)<br>• Decreased pain sensation (95)<br>• Behavioral problems (self-destructive) (95)<br>• Autistic-like behavior (95)<br>• Sleep disturbances (97)<br>• Hearing problems (98)<br>• Cleft palate (95)

			• Urinary tract abnormalities (96)<br>• Dental problems (95)<br>• Hypertrichosis (95)<br>• Limb abnormalities (95)<br>• Visual problems (95)<br>• Abnormalities of the external genitalia (95)<br>• Feeding problems (99)
Costello (like) syndrome	• Growth hormone deficiency (100)<br>• Hypothyroidism (100)<br>• Delayed or dysregulated puberty (100)<br>• Hyperprolactinemia (101)<br>• Parathyroid adenoma (101)<br>• Hyperinsulinemic hypoglycemia (100)	• Increased risk of malignancy (100)<br>• Cardiac problems (100)<br>• Obstructive sleep apnea<br>• Respiratory problems (100)<br>• Pyloric stenosis (100)<br>• Gastroesophageal reflux (100)	• Intellectual disability (100)<br>• Hypotonia (100)<br>• Neurologic abnormalities (100)<br>• Failure to thrive (100)<br>• Epilepsy (102)<br>• Behavioral/social problems (100)<br>• Dermatologic abnormalities (100)<br>• Musculoskeletal abnormalities (100)<br>• Dental abnormalities (100)<br>• Visual problems (100)
Cri-du-Chat syndrome		• Cardiac abnormalities (103)<br>• Renal abnormalities (103)	• Severe psychomotor retardation, including intellectual disability (104)<br>• Microcephaly (104)<br>• Renal abnormalities (103)<br>• Behavioral problems (self-injurious behavior) (105)<br>• Autism spectrum disorder (106)<br>• Scoliosis (105)<br>• Hypotonia (103)<br>• Myopia and cataract (103)<br>• Cryptorchidism (103)<br>• Hypospadias (103)
CTNNB1 syndrome (NEDSDV syndrome)		• Vomiting and constipation (107)<br>• Respiratory infections (107)	• Intellectual disability (108)<br>• Behavioral problems (aggression, automutilation, fecal smearing) (108)<br>• Autistic-like behavior (109)<br>• Microcephaly (109)<br>• Motor delay (109)

			• Visual problems (109)<br>• Distal spasticity/hypertonia (107)<br>• Truncal hypotonia (107)<br>• ADHD (107)<br>• Scoliosis (107)<br>• Hip dysplasia (107)
Dandy-Walker syndrome	• Growth retardation (110)<br>• Fetal growth restriction (111)	• Congenital heart disease (111)<br>• Polycystic kidneys (111)	• Congenital intracranial malformation compromising a spectrum of brain abnormalities (110)<br>• Psychomotor retardation, including intellectual disability (110)<br>• Hypotonia (110)<br>• Scoliosis (110)<br>• Visual problems (110)<br>• Facial clefts (111)<br>• Limb and abdominal wall abnormalities (111)<br>• Diaphragmatic hernia (111)<br>• Ambiguous genitalia (111)
DiGeorge syndrome (22q11.2 deletion)	• Hypocalcemia (112,113)<br>• Hypoparathyroidism (114)<br>• Hypothyroidism (115)<br>• Hyperthyroidism (115)<br>• Growth hormone deficiency (116)<br>• Obesity (115)	• Gastroesophageal reflux (117)<br>• Constipation (117)<br>• Vomiting (117)<br>• Thrombocytopenia (115)<br>• Thymic hypoplasia (115)<br>• Recurrent infections (e.g. pneumonia, otitis media) (115)<br>• Rhabdoid tumor (118)<br>• Cardiac abnormalities (115)	• Intellectual disability (119)<br>• Hemiparesis (115)<br>• Hypotonia (120)<br>• Camptodactyly (121)<br>• Seizures (122)<br>• ADHD (123)<br>• Autistic spectrum disorder (124)<br>• Neuropsychiatric manifestations (124,125)<br>• Sleep disturbances (126)<br>• Visual problems (115)<br>• Hearing problems (115)<br>• Scoliosis (115)<br>• Cleft palate (127)<br>• Urogenital problems (115)
Disorders of Sex Development[a]	• Subfertility/infertility (128)<br>• Cortisol deficiency (128)	• Electrolyte disorders (128)<br>• Hypotension (128)	• Ambiguous genitalia (128)

	• Disturbed androgen synthesis (128)<br>• Failure to thrive (128)	• Renal, adrenal, and lung dysgenesis (only for loss-of-function mutations in WNT4) (129)<br>• Congenital heart disease (especially for loss-of-function mutations in GATA4) (128)	• Campomelic dysplasia (only for loss-of-function mutations in SOX9) (128)<br>• Psychosocial problems (130)
Down syndrome (trisomy 21)	• Hypogonadism (131)<br>• (Subclinical) hypothyroidism (132)<br>• Increased risk of type I diabetes (132)<br>• Osteopenia (133)<br>• Osteoporosis (133)<br>• Overweight/obesity (133)<br>• Short stature (132)	• Overall risk for autoimmune diseases (132)<br>• Sleep apnea (133)<br>• Congenital heart disease (134)<br>• Gastrointestinal problems (133)	• Intellectual disability (135)<br>• Hearing impairment (133)<br>• Attentional impairment (136)<br>• Early onset of Alzheimer disease (135)<br>• Visual problems (133)<br>• Behavioral and psychological problems (133)<br>• Seizures (133)
Hypogonadotropic hypogonadism with anosmia (Kallmann syndrome)	• Hypogonadotropic hypogonadism (137)<br>• Advanced skeletal maturation (137)<br>• Obesity (138)<br>• Short stature (137)	• Renal agenesis (137)	• Hyposmia/anosmia (139)<br>• Cleft palate (137)<br>• Hearing impairment (137)<br>• Visual problems (138)<br>• Psychological problems (137)<br>• Low muscle mass (137)<br>• Agenesis of corpus callosum (138)
Hypogonadotropic hypogonadism without anosmia (Kiss1R mutation)	• Hypogonadotropic hypogonadism (140)<br>• Growth hormone deficiency (141)<br>• Impaired cortisol secretion (141)		
Jacobsen syndrome (11q terminal deletion syndrome)	• Growth hormone deficiency (142)<br>• Hypothyroidism (142)<br>• Growth retardation (143)	• Constipation (143)<br>• Abnormal platelet function, thrombocytopenia or pancytopenia (143)<br>• Deficit of cellular or humoral immunity with low IgM and IgA (144,145)<br>• Increased risk of malignancy (143)	• Intellectual disability (143)<br>• Behavioral problems (143)<br>• Genital, central nervous system and skeletal malformations (143)

		• Cardiovascular, renal, and gastrointestinal tract malformations (143)	
Joubert syndrome	• Central diabetes insipidus (146)<br>• Diabetes mellitus type 1 (146)<br>• Pubertas praecox (146)<br>• Hypothyroidism (146)<br>• Growth hormone deficiency (146)<br>• Panhypopituitarism (146)	• Hypertension (147)<br>• Kidney disease (147)<br>• Alternating tachypnea and/or apnea (148)<br>• Elevated transaminases and gamma-glutamyl transferase (146)<br>• Gastrointestinal complications (146)<br>• Congenital heart disease (146)	• Intellectual disability (148)<br>• Hypotonia (148)<br>• Brain abnormalities (146)<br>• Ataxia (146)<br>• Visual problems (146)<br>• Scoliosis (146)
JS-X syndrome			• Conductive and sensorineural hearing loss (149)<br>• Underdeveloped shoulder musculature (149)<br>• Outer and middle ear deformity (149)<br>• Laryngeal obstruction (149)
Kabuki syndrome	• Growth hormone deficiency (150)<br>• Obesity (151)<br>• Hypothyroidism (151)<br>• Hypoglycemia (150)<br>• Delayed sexual development (150)<br>• Pubertas praecox (150)<br>• Diabetes insipidus (150)	• Gastroesophageal reflux (152)<br>• Immune dysfunction (150)<br>• Congenital heart defects (150)<br>• Gastrointestinal malformations (150)<br>• Renal abnormalities (150)<br>• Malignancies (150)	• Intellectual disability (150)<br>• Autism-spectrum disorder (150)<br>• Ocular abnormalities (152)<br>• Hearing deficit (150)<br>• Joint hypermobility (153)<br>• Hypotonia (150)<br>• Seizures (150)<br>• Scoliosis (154)<br>• Genitourinary abnormalities (150)<br>• Feeding problems (150)<br>• Microcephaly (150)

KAT6A syndrome		• Cardiac abnormalities (155)<br>• Gastrointestinal complications, including reflux and constipation (155)<br>• Infections (155)	• Intellectual disability (155)<br>• Developmental delay (155)<br>• Microcephaly (155)<br>• Visual problems (155)<br>• Behavioral problems (155)<br>• Sleep problems (155)<br>• Hypotonia (156)<br>• Cryptorchidism (156)<br>• Syndactyly (156)
Klinefelter syndrome	• Diabetes mellitus type II (157)<br>• Obesity (158)<br>• Hypogonadism (159)<br>• Osteoporosis (159)<br>• Gynaecomastia (160)<br>• Insulin resistance (157)<br>• Metabolic syndrome (157)<br>• Dyslipidemia (161)<br>• Short stature (160)	• Liver adenoma (162)<br>• Increased risk of male breast cancer (163)<br>• Cardiovascular problems (161)	• Genital abnormalities (160)<br>• Neurocognitive disorders (160)<br>• Psychosocial problems (160)
L1CAM mutation	• Growth hormone deficiency (164)	• Hirschsprung disease (165)<br>• Dysphagia (164)<br>• Constipation (164)<br>• Cardiac malformations (164,166)	• Hydrocephalus (165)<br>• Intellectuality disability (165)<br>• Motor delay (166)<br>• Spasticity of the legs (165)<br>• Corpus callosum hypogenesis/agenesis (165)<br>• Arthrogryposis (164)<br>• Scoliosis (164)
Myhre syndrome	• Abnormal onset of puberty (167)<br>• Advanced skeletal maturation (167)<br>• Short stature (168)	• Hypertension (167)	• Intellectual disability (168)<br>• Behavioral problems (169)<br>• Deafness (169)<br>• Restrictive joint movement (168)<br>• Thick calvarium (168)

Neurofibromatosis type 1	• Increased risk of pheochromocytoma (170)<br>• Growth hormone deficiency (170)<br>• Osteopenia/osteoporosis (170)<br>• Central precocious puberty[b] (171)<br>• Obesity[b] (171)<br>• Impaired glucose intolerance[b] (171)<br>• Growth hormone excess[b] (171)<br>• ACTH deficiency[b] (171)<br>• Hypogonadotropic hypogonadism[b] (171)<br>• Thyrotropin deficiency[b] (171)<br>• Hyperparathyroidism (172)	• Hypertension (173)<br>• Hypovitaminosis D (174)<br>• Increased risk of malignancy (170)<br>• Vasculopathy (170)	• Visual problems (175)<br>• ADHD (176)<br>• Autistic-like behavior (170)<br>• Behavioral and learning problems (170)<br>• Psychosocial problems (170,177)<br>• Depression (177)<br>• Plexiform neurofibromas (170)<br>• Sleep disturbances (178)<br>• Increased risk of stroke (179)<br>• Scoliosis (170)<br>• Dermatologic problems (170)<br>• Brain tumors (170)
Noonan syndrome	• Male gonadal dysfunction (180)<br>• Hypothyroidism (180)<br>• Short stature (180)	• Thrombocytopenia (181)<br>• Leukemia (182)<br>• Congenital heart defects (180)<br>• Renal abnormalities (180)	• Intellectual disability (180)<br>• ADHD (181)<br>• Ocular abnormalities (181)<br>• Hearing loss (181)<br>• Pectus excavatum or carinatum (181)<br>• Scoliosis (181)<br>• Feeding difficulties (181)<br>• Urinary tract abnormalities (182)<br>• Lymphedema (181)<br>• Dental problems (181)<br>• Genital abnormalities (181)
PNPLA6 gene mutation	• Anterior hypopituitarism (growth hormone, thyroid hormone, or gonadotropin deficiencies) (183)<br>• Short stature (183)		• Cerebellar ataxia (183)<br>• Spasticity (183)<br>• Visual problems (183)<br>• Peripheral neuropathy (183)<br>• Hair abnormalities (183)<br>• Impaired cognitive functioning (183)
PTEN hamartoma tumor syndrome	• Goiter of thyroid (184)<br>• Obesity (185)<br>• Increased insulin sensitivity (185)	• Hamartomatous tumors (184)<br>• Increased risk of malignancies (184)<br>• Vascular malformations (186)	• Macrocephaly (184)<br>• Developmental delay, including intellectual disability (184)<br>• Myopathy (184)<br>• Joint hyperextensibility (184)<br>• Pectus excavatum (184)

			• Scoliosis (184)<br>• Autism spectrum disorder (184)<br>• Genitourinary malformations (186)<br>• Dermatologic problems (186)
Prader-Willi (like) syndrome	• Obesity (187)<br>• Growth hormone deficiency (187)<br>• Hypogonadism (18,187)<br>• Hypothyroidism (18,188)<br>• Diabetes mellitus type II (18,187)<br>• Central adrenal insufficiency (22)<br>• Hypothalamic dysfunction (187)<br>• Osteoporosis (187)<br>• Short stature (187)	• Hypercholesterolemia (18,189)<br>• Hypertension (18)<br>• Hypovitaminosis D (18,190)<br>• Peripheral edema (191)<br>• Constipation (192)<br>• Sleep apnea (18)	• Intellectual disability (187)<br>• Sleep disturbances (187)<br>• Behavioral problems and self-injurious behavior (193)<br>• High pain threshold (187)<br>• Inability to vomit (187)<br>• Ocular abnormalities (187)<br>• Dental problems (194)<br>• Scoliosis (18,187)<br>• Heart failure (195)<br>• Genital abnormalities (187)<br>• Psychiatric problems, such as psychotic illness (196)<br>• Autistic features (187)<br>• Hypotonia (187)
Rett syndrome	• Osteopenia (197)	• Gastroesophageal reflux (197)<br>• Constipation (198)<br>• Gallbladder disease (199)<br>• Hyperventilation (200)<br>• Cardiovascular autonomic dysregulation (200)	• Intellectual disability (197)<br>• Disturbed motor control (197)<br>• Seizures (199)<br>• Parkinsonian features (199)<br>• Dystonia (197)<br>• Hypotonia (198)<br>• Sleep disturbances (198)<br>• Behavioral problems (199)<br>• Autistic features (198)<br>• Psychosocial problems (199)<br>• Scoliosis (201)<br>• Difficulty to swallow (199)
Ring chromosome 21	• Short stature (202)	• Thrombocytopenia (203)<br>• Association with myelodysplasia, leukemia and lymphomas (203)<br>• Immunodeficiency (203)	• Intellectual disability (203)<br>• Motor delay (203)<br>• Hypertonia (203)<br>• Seizures (203)<br>• Microcephaly (203)

		• Cardiomyopathy (202)	
Saethre-Chotzen syndrome	• Short stature (204)	• Renal abnormalities (205)<br>• Congenital heart defects (204)<br>• Obstructive sleep apnea (204)	• Intellectual disability (205)<br>• Hearing loss (206)<br>• Intracranial hypertension (206)<br>• Various skeletal findings (204)<br>• Cleft palate (204)<br>• Strabismus (205)
Say-Barber-Biesecker-Young-Simpson syndrome (KAT6B mutation)	• Thyroid abnormalities/thyroid dysfunction (207,208)	• Congenital heart disease (209)	• Intellectual disability (209)<br>• Hypoplastic teeth (209)<br>• Hearing impairment (209)<br>• Cleft palate (209)<br>• Genital and patellar abnormalities (207)<br>• Feeding difficulties (207)<br>• Dental abnormalities (207)<br>• Visual problems (208)
Sifrim-Hitz-Weiss syndrome	• Hypogonadism (210)<br>• Growth hormone deficiency (211)	• Chronic renal insufficiency (210)<br>• Heart defects (210)	• Developmental delay (210)<br>• Intellectual disability (210)<br>• Macrocephaly (210)<br>• Hypotonia (211)<br>• Brain abnormalities (210)<br>• Hearing loss (210)<br>• Ocular abnormalities (210)<br>• Skeletal and limb abnormalities (210)
Silver-Russell syndrome	• Growth hormone deficiency (104)<br>• Hypoglycemia (104)<br>• Delayed bone age (212)<br>• Short stature (212)	• Gastroesophageal reflux (212)<br>• Constipation (213)<br>• Vomiting (213)<br>• Cardiac defects (104)<br>• Wilms' and other tumors (104)	• Hypotonia (214)<br>• Joint problems (215)<br>• Scoliosis (214)<br>• Urogenital abnormalities (216)<br>• Inguinal hernia (217)<br>• Excess sweating (212)<br>• Developmental delay (212)

Smith-Lemli-Opitz syndrome	• Adrenal insufficiency (218)<br>• Hypo-aldosteronism (219)<br>• Hypothyroidism (218)	• Gastroesophageal reflux (220)<br>• Constipation (218)<br>• Gastro-intestinal motility problems (218)<br>• Hypocholesterolemia (221)<br>• Immune deficiency (218)<br>• Electrolyte abnormalities (218)<br>• Congenital heart defects (218)	• Intellectual disability (218)<br>• Psychiatric problems (222)<br>• Sleep disturbance (222)<br>• Ocular abnormalities (218)<br>• Hearing loss (220)<br>• Congenital dislocation of the hip (223)<br>• Short limbs (218)<br>• Genital abnormalities (218)<br>• Cleft palate (218)<br>• Skeletal problems (218)
Smith-Magenis syndrome	• Obesity (224)<br>• Short stature (104)	• Renal abnormalities (224)<br>• Congenital cardiac defects (104)	• Intellectual disability (224)<br>• Developmental delay (224)<br>• Seizures (224)<br>• Hearing loss (224)<br>• Ocular abnormalities (224)<br>• Cleft palate (224)<br>• Sleep disturbances (224)<br>• Challenging behavior and self-injurious behaviors (224)<br>• Skeletal abnormalities (104)<br>• Decreased pain sensitivity (104)
Sotos-like syndrome	• Overgrowth (225)<br>• Advanced bone age (226)	• Gastroesophageal reflux (226)<br>• Cardiac abnormalities (226)<br>• Increased risk of tumors (226)<br>• Renal abnormalities (227)	• Intellectual disability (225)<br>• Delayed motor development (226)<br>• Hypotonia (228)<br>• Feeding difficulties (226)<br>• Macrocephaly (226)<br>• Scoliosis (226)<br>• Genitourinary abnormalities (226)<br>• Seizures (226)
Tatton-Brown-Rahman syndrome	• Overgrowth (229)<br>• Obesity (230)	• Autonomic dysfunction, including central sleep apnea and orthostatic hypotension (229)<br>• Cardiac defects (229)	• Intellectual disability (229)<br>• Hypotonia (229)<br>• Brain abnormalities (229)<br>• Skeletal abnormalities (229)<br>• Joint hypermobility (229)

		• Gastrointestinal problems (229)<br>• Possibly increased risk of tumors (229)	• Behavioral/psychiatric problems, including autistic spectrum disorder and ADHD (229,230)<br>• Seizures (230)
TBL1X mutation	• Central hypothyroidism (231)<br>• Obesity (231)	• Constipation (232)	• Autistic spectrum disorder (233)<br>• Hearing loss (231)<br>• ADHD (232)<br>• Chiari malformation type I (232)
Tetra X syndrome (48,XXXX)	• Premature ovarian failure (234)<br>• Pituitary hormone deficiencies due to a Rathke's cleft cyst (235)<br>• Osteoporosis (234)	• Hemolytic anemia (236)<br>• Thrombocytopenia (236)<br>• Systemic lupus erythematosus (236,237)	• Intellectual disability (234,238)<br>• Sleep disturbances (hypersomnia) (239)<br>• Alopecia (236)<br>• Skeletal abnormalities (238)
Triple X syndrome (47,XXX)	• Premature ovarian failure (240)<br>• Tall stature (240)	• Abdominal pain (241)<br>• Renal abnormalities (240)	• Hypotonia (240)<br>• Seizures (240)<br>• Genitourinary abnormalities (240)<br>• Cognitive defects and learning disabilities (240)<br>• Various psychological problems (attention deficit; mood disorders) (240)
TRPV4 mutation	• Osteoporosis (242)<br>• Short stature (242)	• Respiratory dysfunction (242)	• Neuromuscular problems (242)<br>• Skeletal dysplasias (242)<br>• Sensorineural hearing loss (242)
Tuberous sclerosis complex	• Increased risk of neuroendocrine tumors (rare) (243)	• Renal abnormalities (243)<br>• Pulmonary problems (243)<br>• Increased risk of tumors (243)<br>• Cardiac lipoma (244)<br>• Gastrointestinal problems (243)	• Epilepsy/seizures (243)<br>• Intellectual disability (243)<br>• TAND (243)<br>• Retinal hamartomas (243)<br>• Dermatological problems (243)

Turner syndrome	• Hypothyroidism (245)<br>• Hyperthyroidism (245)<br>• Hypogonadism (245)<br>• Obesity (245)<br>• Osteopenia or osteoporosis (245)<br>• Glucose intolerance (245)<br>• Diabetes mellitus type II (245)<br>• Infertility/subfertility (245)<br>• Delayed bone age (245)<br>• Short stature (245)	• Hypercholesterolemia (245)<br>• Hypertension (246)<br>• Celiac disease (245)<br>• Elevated hepatic enzymes (245)<br>• Increased risk of auto-immune disease (245)<br>• Inflammatory bowel disease (245)<br>• (Congenital) heart defects (245)<br>• Renal abnormalities (245)	• Psychological problems (anxiety, depression)(245)<br>• Visual problems(245)<br>• Hearing problems(245)<br>• Developmental problems (motoric, cognitive and psychosocial) (245)<br>• Dermatological problems (245)<br>• Dental problems (245)<br>• Lymphedema of hands and feet (245)<br>• Scoliosis (245)
Williams-Beuren syndrome	• Osteopenia/osteoporosis (247)<br>• Hypercalcemia (247)<br>• Hypothyroidism (247)<br>• Obesity (248)<br>• Growth hormone deficiency (249)<br>• Diabetes mellitus (247)<br>• Early onset of puberty (247)<br>• Short stature (247)	• Hypertension (247)<br>• Gastroesophageal reflux (247)<br>• Constipation (247)<br>• Celiac disease (247)<br>• Recurrent otitis media (247)<br>• Cardiovascular disease (247)<br>• Abdominal pain (247)<br>• Diverticular disease (247)	• Intellectual disability (247)<br>• Epilepsy (250)<br>• Sleep dysregulation (247)<br>• ADHD (247)<br>• Ocular abnormalities (247)<br>• Hearing loss (247)<br>• Hypotonia (247)<br>• Scoliosis or lordosis (247)<br>• Joint laxity (247)<br>• Genitourinary problems (247)<br>• Hyperacusis (247)<br>• Increased risk of stroke (247)<br>• Dental problems (247)<br>• Neurological problems (247)
45,X/46,XY mixed gonadal dysgenesis	• Autoimmune thyroid disease (251)<br>• Delayed puberty (251)<br>• Short stature (251)	• Renal malformations (251)	• Atypical genitalia (251)<br>• Intellectual disability (252)
48,XXXY syndrome	• Hypogonadism (253)<br>• Hypothyroidism (253)<br>• Diabetes mellitus type II (253)<br>• Osteoporosis (253)<br>• Tall stature (253)	• Asthma (253)<br>• Allergies (253)<br>• Cardiac abnormalities (253)<br>• Deep vein thrombosis (253)<br>• Constipation (253)<br>• Obstructive sleep apnea (253)	• Intellectual disability (253)<br>• Speech and motor delays (253)<br>• Small testicular size (253)<br>• Tremor (253)<br>• Scoliosis (253)<br>• Seizures (253)

48,XXYY syndrome	• Hypogonadism (254)<br>• Diabetes mellitus type II (255)<br>• Hypothyroidism (255)<br>• Osteoporosis (253)<br>• Tall stature (255)	• Allergies (255)<br>• Asthma (255)<br>• Constipation (255)<br>• Gastroesophageal reflux (255)<br>• Cardiac abnormalities (255)<br>• Deep vein thrombosis (255)<br>• Renal dysplasia (253)<br>• Obstructive sleep apnea (253)	• Intellectual disability (253)<br>• Speech and motor delays (253)<br>• Autism spectrum disorder or deficits in social communication or interaction skills (256)<br>• Psychological problems (255)<br>• Scoliosis (255)<br>• Seizures (255)<br>• Tremors (254)<br>• Cryptorchidism (255)<br>• Brain abnormalities (255)<br>• Tremor (253)

Abbreviations: ACTH, adrenocorticotropic hormone; ADHD, attention deficit hyperactivity disorder; TAND, TSC-associated neuropsychiatric disorders, e.g. autism, anxiety, depression, aggressive behavior, sleep disorders
In reality, some manifestations might have a similar prevalence as in the general population, because of publication bias. Although the literature was thoroughly searched, the overview might be incomplete.
[a] There are many different types of Disorders of Sex Development. Therefore, we advise to check the specific type of disorder in the literature for the specific clinical manifestations. [b] These manifestations are mostly associated with optic pathway gliomas in patients with Neurofibromatosis type 1.
